# Influence of Neonatal Exposure to Hyperoxia on Skeletal Muscle in a Rat Model

**DOI:** 10.3390/pediatric17060125

**Published:** 2025-11-14

**Authors:** Kentaro Awata, Irena Santosa, Yoshiteru Arai, Mayu Nakagawa, Hiroki Suganuma, Hiromichi Shoji

**Affiliations:** 1 Department of Pediatrics and Adolescent Medicine, Graduate School of Medicine, Juntendo University, Bunkyo, Tokyo 113-8421, Japan; k-awata@juntendo.ac.jp (K.A.); yo-arai@juntendo.ac.jp (Y.A.); mnakaga@juntendo.ac.jp (M.N.); hsuganu@juntendo.ac.jp (H.S.); hshoji@juntendo.ac.jp (H.S.); 2 Department of Pediatrics, Faculty of Medicine, Juntendo University, Bunkyo, Tokyo 113-8421, Japan

**Keywords:** preterm birth, hyperoxia, fiber type, skeletal muscle, muscle atrophy

## Abstract

**Background/Objectives**: Premature births below 32 weeks of gestation generally require respiratory oxygen support, leading to a relatively hyperoxic environment compared to in utero conditions. Transient hyperoxia exposure has been linked to an elevated risk of chronic lung disease and retinopathy of prematurity; however, its effects on skeletal muscles remain elusive. This study aimed to investigate the effects of hyperoxic exposure in rats as a model of premature infants receiving supplemental oxygen (30–60% O_2_ for several weeks). We hypothesized that rats exposed to postnatal hyperoxia would exhibit muscle fiber atrophy and alterations in fiber type. **Methods**: We used a rat model in which newborns were exposed to 80% oxygen from birth until postnatal day 12. We assessed the gastrocnemius muscles of rat legs at 12 weeks. **Results**: Rats exposed to hyperoxia showed substantially increased protein expression of Atrogin-1, along with elevated levels of adipophilin, myogenic differentiation factor 1, and myogenin. No significant changes were observed in the expression of slow or fast myosin heavy chain proteins. However, myofiber size in the gastrocnemius muscle was reduced in the hyperoxia-exposed group compared to the control group. **Conclusions**: Thus, transient hyperoxia exposure during early life can impede skeletal muscle development, potentially extending into adulthood.

## 1. Introduction

Improved perinatal care has enhanced the survival rates of infants born preterm, even those born between 28–31 weeks. Very preterm infants are at a greater risk than full-term infants for suffering any negative effects on the pulmonary and cardiovascular systems because of organ immaturity and their management (specifically supplemental oxygen), which can significantly impact them in the long term and their quality of life, potentially resulting in death. Furthermore, prematurity has been associated with disruptions in the uptake of amino acids by skeletal muscle, which may adversely affect muscle development and growth. These alterations can contribute to a higher risk of metabolic disorders, including obesity and type 2 diabetes, both of which are frequently observed in individuals born very preterm [1].

Fetal environment in utero is relatively hypoxic compared with the extrauterine environment and largely depends on the oxygen partial pressure gradient between the maternal blood (uterine artery/placenta) and fetal blood (umbilical vein). A positive partial pressure gradient drives oxygen. It diffuses passively from the maternal arterial blood into the placental intervillous space, where fetal hemoglobin binds it with a high oxygen affinity [2]. At birth, the initiation of breathing and lung expansion markedly increase oxygen availability to tissues, resulting in a physiological rise in oxidative stress. Because lung structure, function, and antioxidant defenses mature mainly during late gestation, very preterm infants—with their immature lungs, compliant thoracic cages, and underdeveloped antioxidant systems—often require immediate resuscitation and supplemental oxygen to achieve postnatal stabilization. In many cases, oxygen therapy must continue for extended periods ranging from minutes to days, weeks, or even months. However, prolonged or excessive oxygen exposure can induce oxidative stress, damaging cellular proteins, lipids, and DNA, and potentially causing hyperoxia-related injury. Such oxidative insults can disrupt normal muscle development, alter muscle fiber composition, and impair contractile function [3]. Moreover, oxygen supplementation after birth has been shown to influence epigenetic regulation, with possible long-term effects on growth and development [2].

Intramuscular triglycerides accumulate as lipid droplets decorated with proteins such as perilipin. In an animal model of disuse-induced muscle atrophy, we observed an increase in Plin2 and a corresponding decrease in Plin5 expression. Furthermore, the increased Plin2 expression is associated with increased expression of muscle atrophy-related genes, such as MuRF-1, Atrogin, and p53 [4].

Skeletal muscles undergo a crucial developmental phase during the late second and the beginning of the third trimesters of gestation [3]. Myofibers are involved in fiber type determination, growth, and maturation of excitation–contraction coupling and bioenergetic systems [5]. Premature exposure of developing skeletal muscles to high oxygen levels may induce long-lasting alterations in function, as observed in other organs [5]. The effects of hyperoxia on skeletal muscles remain elusive despite the indirect evidence of skeletal muscle defects. Thus, this study aimed to show the effects of hyperoxia on muscle growth, muscle atrophy, and fiber typing, focusing on those born at 28–31 weeks of gestation and who receive supplemental oxygen.

## 2. Materials and Methods

The Animal Care Committee of Juntendo University, Tokyo, Japan, approved the study protocol (Approval No.: 1494). Isoflurane was administered to induce deep anesthesia, and efforts were made to minimize suffering.

### 2.1. Animals

Pregnant Sprague Dawley (SD) rats (8–10 weeks old; Sankyo Labo Service Corporation, Inc., Tokyo, Japan) were individually housed in cages with ad libitum access to food and water and were exposed to 12 h light/dark cycles at 24–25 °C. Two dams were allowed to give birth spontaneously (10 pups for each dam, 6M/4F in the HO group, and 5M/5F in the control group). After giving birth, the pups were maintained together with their dam in either 80% O_2_ (a mixture of medical-grade 100% O_2_ and room air; Oxycycler ProOx 110; Biospherix, Lacona, NY, USA) or room air from postnatal day (PD) 0 to 12. After day 12, both the pups from each group were housed together with their dams until week 3 in the room air. After three weeks, the pups were weaned from their dams, and we selected five male pups with body weight closest to the average from each group to grow in room air until they were 12 weeks old. No mortality occurred during the period of transient neonatal hyperoxia exposure, and all 5 male pups were retained for inclusion in the experimental protocol.

### 2.2. Experimental Groups

**Figure 1:**
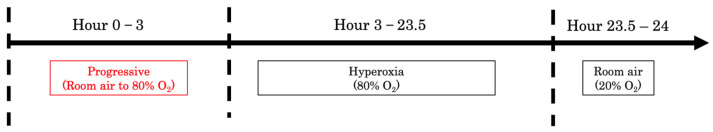
Oxygen cycle in the hyperoxia (HO) group from PD 0 to PD 12. The litter, together with the dam, was exposed to high-oxygen levels from PD 0 to PD 12. Every day, the litter and the dam were allowed to be brought back to room air for 30 min and then put back to 80% of oxygen progressively in 3 h.

One litter was designated as the control group, and the other as the hyperoxia (HO) group. The litter assigned to the HO group was maintained in an environment with 80% O_2_, beginning on PD 0. Each day, the chamber was opened for 30 min, allowing the oxygen concentration to return to ambient room-air levels. Then, the oxygen concentration was gradually increased back to 80% over a 3 h period using an oxycycler. For the rest of the day, the pups, together with the dam, stayed in an 80% O_2_ environment. This daily exposure cycle was continued from PD 0 to PD 12 (Figure 1). The litters of the control group stayed in the room air with their dam. From PD 13 until week 12, the pups in both groups were raised in room air (21% O_2_). Body weight was measured at three time points: day 12, week 4, and week 12. At week 12, five male pups from each group were dissected. The gastrocnemius muscles were collected, flash-frozen in liquid nitrogen, and fixed in 10% formalin.

### 2.3. Histology

Harvested gastrocnemius muscles fixed in 10% formalin were embedded in paraffin blocks and cut into 4 μm-thick sections. The slides were stained with hematoxylin and eosin. They were subsequently observed under an inverted microscope (Nikon Eclipse Ci-L; Nikon Instruments Inc., Melville, NY, USA) connected to a digital camera (DS-L3; Nikon, Tokyo, Japan) at 10×. Three images from different fields of view were captured from each slide, and the fiber diameters were measured from these three fields. Five measurements were recorded using ImageJ (1.53a; National Institute of Health, Bethesda, MD, USA) and averaged to determine the muscle fiber cross-section.

### 2.4. Western Blot Analysis

Gastrocnemius muscles were homogenized using radio-immunoprecipitation assay buffer that contained 50 mmol/L Tris-HCl buffer (pH 7.6), 150 mmol/L NaCl, 1% Nonidet^®^ P40, 0.5% sodium deoxycholate, a protease inhibitor cocktail, and 0.1% sodium dodecyl sulfate (Nacalai Tesque, Kyoto, Japan). A 10 μg protein concentration per sample was resolved with NuPAGE™ LDS Sample buffer (Thermo Fisher Scientific, Inc., Danvers, Waltham, MA, USA), which was then transferred into polyvinylidene fluoride membranes, and blocked in Bullet Blocking One for Western Blotting (Nacalai Tesque, Kyoto, Japan) for 10 min. Subsequently, the membranes were exposed to primary antibodies: Anti-TRIM63 (Proteintech, #55456-1-AP), Anti-FBX-32 (Abcam, #ab168372), Anti-Catalase (Abcam, #ab52477), Anti-Slow Skeletal Myosin Heavy Chain (Abcam, #ab11083), Anti-Fast Myosin Skeletal Heavy Chain (Abcam, #ab91506), Anti-Upload -81), Anti-myogenic differentiation factor 1 (MyoD) (Santa Cruz Biotechnology, #SC-377460), Anti-Myogenin (Santa Cruz Biotechnology, #SC-52903), and incubated at 4 °C overnight. After washing three times with Tris-buffered saline containing 0.1% Tween-20 (TBST), the membranes were incubated with horseradish peroxidase-conjugated goat anti-rabbit IgG (Cell Signaling Technology, Danvers, MA, USA) for 1 h at 24 °C with gentle shaking. The membranes were subsequently washed four times with TBST, after which the blots were developed using ImmunoStar LD (FUJIFILM Wako Pure Chemical Corporation, Osaka, Japan). Band intensities were quantified using Fusion software (version 1.51; National Institutes of Health, Bethesda, MD, USA).

### 2.5. Statistical Analysis

Statistical differences were analyzed using Student’s *t*-test. All data were analyzed using GraphPad Prism Ver.9 (GraphPad Software Inc., San Diego, CA, USA). Statistical significance was set at *p* < 0.05.

## 3. Results

### 3.1. Body Weight and Body Weight Gain

**Figure 2:**
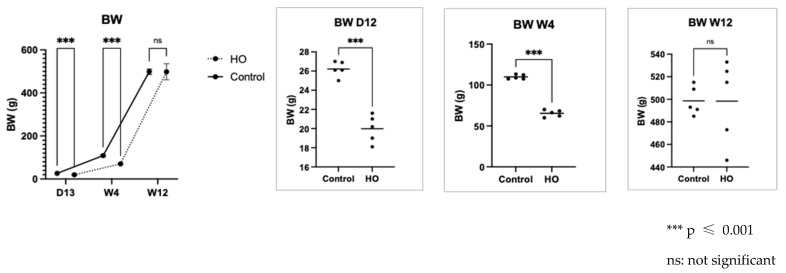
Body weight at three time points. Body weights of the offspring in the hyperoxia (HO) and control groups were recorded on day 12, week 4, and week 12. HO offspring had significantly reduced body weights on day 12 and week 4 compared to the control group offspring.

Body weight was measured at three time points to evaluate muscle atrophy: day 12, week 4, and week 12 (Figure 2). On day 12, the pups exposed to neonatal hyperoxia were substantially smaller than those in the control group. These findings persisted until week 4. However, no significant differences in weight were observed between the two groups by week 12 (Figure 2).

### 3.2. Impact of Transient Neonatal Hyperoxia on Muscle Atrophy and Myogenic Factor Expression

**Figure 3:**
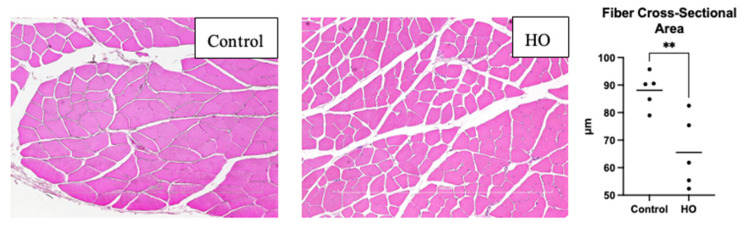
HE Stain and Fiber Cross-Sectional Area. Representative HE stain images from the HO and control groups. The size of each fiber in the HO groups was smaller, and the gap between fibers in the HO group was bigger. The fiber cross-sectional area was smaller in the HO group compared to the control group (** *p* < 0.001).

**Figure 4:**
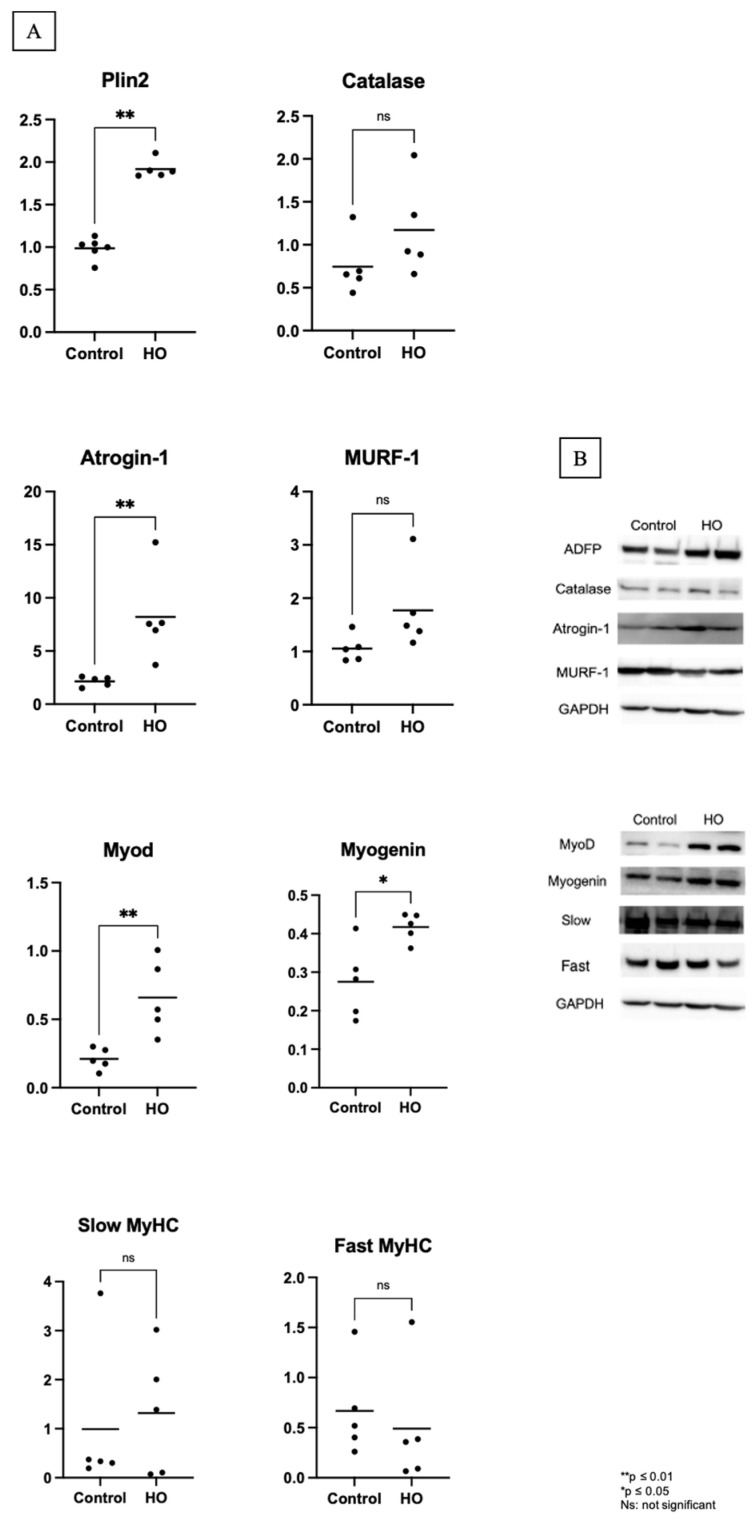
Protein expression from Western blot of the gastrocnemius muscle. (**A**) Protein expression from Western blotting of Plin2, catalase, Atrogin-1, MuRF-1, MyoD, myogenin, and slow and fast MyHC. N = 5 in both groups. Plin2, Atrogin-1, MyoD, and myogenin protein levels were higher in the hyperoxia (HO) group than in the control group (** *p* ≤ 0.01; * *p* ≤ 0.05; ns: not significant). (**B**) The representative bands from the Western blot for each protein, with GAPDH as the internal standard.

Light microscopic examination of the gastrocnemius muscle showed decreased fiber cross-sectional areas in the HO group, indicating atrophy (Figure 3). To elucidate the mechanisms underlying muscle atrophy induced by transient neonatal exposure to elevated oxygen levels, we evaluated the expression of Atrogin-1 and MuRF-1. These two E3 ubiquitin ligases play key roles in muscle protein degradation. Atrogin-1 expression was higher in the HO group than in the control group. However, no differences in MuRF-1 levels were found between the two groups (Figure 4).

MyoD, myogenin, and myosin heavy chain (MyHC) are regulatory factors and biomarkers of various differentiation and myogenesis stages. Increased protein expression levels of MyoD and myogenin were observed without differences in the slow and fast MyHC fiber types between the groups (Figure 4).

### 3.3. Effects of Transient Neonatal Hyperoxia on Oxidative Stress and Lipid Accumulation

To assess the impact of transient neonatal exposure to high oxygen levels on redox status, we measured the activity of catalase, a key antioxidant enzyme that decomposes hydrogen peroxide—a major reactive oxygen species (ROS)—into water and oxygen. No significant differences were observed in the catalase levels between the HO and control groups (Figure 4). We measured the adipophilin/perilipin-2 protein levels to evaluate lipid accumulation in skeletal muscle. A substantially higher protein expression of adipophilin in the HO group was observed (Figure 4).

## 4. Discussion

Compared to the in utero environment, newborns are exposed to significantly higher oxygen levels. Neonatal oxygen supplementation further exacerbates this relatively hyperoxic condition, particularly in preterm infants. Sudden exposure to elevated oxygen concentrations in neonates leads to increased ROS production and inflammation in skeletal muscle, while the antioxidant defense system remains underdeveloped in preterm infants [6].

While this rat model is not a premature model, the developmental stages of vital organs—including the eyes, brain, kidneys, lungs, heart, and skeletal muscle—at birth are comparable to those of a human infant born preterm in the second trimester, as proven in a previous study [3]. Deprez et al. found that newborn rats on day 3 showed the skeletal muscle characteristics of 24-week human fetuses. Also, they found another characteristic that can be found in a very preterm newborn (20–26 weeks), such as the remaining large fibers from primary myogenesis, centro-nucleated fibers, and myofibers scattered in the interstitial tissues [3]. The rodent model of transient neonatal exposure to high oxygen is well-established for simulating conditions associated with prematurity, including bronchopulmonary dysplasia, retinopathy of prematurity, and cardiovascular complications [7]. This model is highly relevant for investigating the impact of preterm birth on skeletal muscle development, as the developmental stage of skeletal muscles in newborn rats corresponds to that of human infants born very preterm. Neonatal rats were exposed to hyperoxic gas to simulate the abrupt increase in partial oxygen pressure (pO_2_) associated with preterm birth [8].

Neonatal exposure to high oxygen levels for 12 days led to lower body weight of pups in the HO group from day 13 until week 4. This result was also observed in a previous rat hyperoxia study, in which the authors found that exposure to hyperoxia decreased the body weight of pups [9]. One of the reasons for the decrease in pup body weight might be the changed lung development and respiratory function, which are often observed in neonatal rats exposed to hyperoxia. Lung injury from hyperoxia exposure can decrease oxygen exchange efficiency and energy availability for growth [10]. This lower body weight finding is consistent with a hyperoxia study using Sprague Dawley rats, which reported that neonatal exposure to high oxygen levels resulted in reduced body weight by postnatal Day 14 [9].

Hyperoxia generates ROS and causes inflammation, which damages cellular structures, including lipids. Cells upregulate lipid droplets to store excess fatty acids, thereby preventing lipotoxicity. One of the main families of lipid droplet coat proteins characterized to date is the perilipin family. Plin2, Plin3, and Plin5 are the primary perilipins in the skeletal muscles [11]. Plin2 expression is linked to muscle atrophy-related genes, such as MuRF-1, Atrogin, and p53 [4]. Similar results were noted in this study; we observed an increase in Plin2 levels in the skeletal muscle, which may explain the increased Atrogin-1 expression.

Studies conducted in vitro have shown that ROS can directly enhance the expression of MuRF-1 and Atrogin-1, as well as their ubiquitin ligase activity. In vivo, various muscle-related conditions—including aging and chronic obstructive pulmonary disease—have been associated with elevated ROS levels, which correlate with increased expression of these ubiquitin ligases. Collectively, these findings indicate that the underdeveloped antioxidant defenses and heightened vulnerability to oxidative stress play a significant role in activating the ubiquitin-proteasome pathway through ROS production. This activation contributes to increased muscle protein breakdown, impaired myofiber regeneration, and inhibition of late-stage myotube differentiation, ultimately promoting muscle atrophy [1,3]. ROS production with an immature antioxidant defense system affected atrogin-1 increase in this study, which suggests that muscle atrophy occurs in the HO group, which is also reflected in the decreased fiber cross-sectional areas observed in this group.

The second and third trimester of pregnancy is a crucial period for skeletal muscle development, which involves the maturation of muscle fiber excitation–contraction coupling and fiber type determination [12]. Four transcription factors (muscle regulatory factors, MRFs) of the MyoD family of basic-helix-loop-helix proteins are central to muscle development and differentiation. The primary role of the MRFs is to determine skeletal muscle lineage specification—Myf5 and MyoD—and to regulate muscle differentiation, primarily through myogenin, while MRF4 exhibits functions associated with both lineage determination and differentiation [13]. Upregulated myogenin in the skeletal muscle regulates MuRF-1 and atrogin-1 expression, which promote muscle proteolysis and atrophy [14]. The increase in myogenin levels observed in this study can lead to atrogin-1 activation, an essential regulatory factor for muscle atrophy, which increases myofibrillar protein degradation and causes muscle shrinkage due to excessive protein breakdown. MyoD expression is upregulated in response to protein breakdown, which in turn increases myogenesis. MyoD and myogenin levels were high; however, the fiber cross-section was lower than that of the control group. This implies that the muscle fibers did not form efficiently, which may indicate a defect in the fusion process.

Increased ROS production is related to muscle fiber atrophy, shifts in fiber type (decreased proportion of type I slow fibers and increased proportion of type II fast-fatigue fibers), and fibrofatty replacement in different pathological conditions [15]. We observed no differences in catalase, suggesting that no differences were found in the oxidative stress levels between the HO and control groups. In previous studies [15,16], changes in fiber type were attributed to excessive oxidative stress; however, this study found no differences in oxidative stress and no fiber type shifting in the skeletal muscles.

As stated before, the rat model in this study was not a premature model, which is one of the limitations of this study. Also, the pups in the hyperoxia group were breastfed from a dam that was exposed to hyperoxia, and we could not measure the impact of hyperoxia on the breastmilk of the dam.

## 5. Conclusions

This study underscores the effects of transient neonatal hyperoxia on skeletal muscle development, especially in the context of preterm birth. Although no alterations were observed in the oxidative stress markers or muscle fiber type composition, other indicators indicated muscle atrophy. The increased Plin2, myogenin, and Atrogin-1 expression suggests that immature antioxidant defenses and elevated ROS drive the hyperoxia-induced activation of muscle proteolytic pathways. The observed decrease in the muscle fiber cross-sectional area supports the presence of impaired myofiber growth and regeneration.

## Data Availability

The original contributions presented in this study are included in the article. Further inquiries can be directed to the corresponding author(s).

## Abbreviations

The following abbreviations are used in this manuscript:

ROS	Reactive oxygen species
SD	Sprague Dawley
PD	Postnatal days
